# Integrating Biophysics in Toxicology

**DOI:** 10.3390/cells9051282

**Published:** 2020-05-21

**Authors:** Giorgia Del Favero, Annette Kraegeloh

**Affiliations:** 1 Department of Food Chemistry and Toxicology, Faculty of Chemistry, University of Vienna, Währinger Straße 38-40, 1090 Vienna, Austria; 2 Core Facility Multimodal Imaging, Faculty of Chemistry, University of Vienna Währinger Straße 38-40, 1090 Vienna, Austria; 3 INM—Leibniz-Institut für Neue Materialien GmbH, Campus D2 2, 66123 Saarbrücken, Germany; Annette.Kraegeloh@leibniz-inm.de

**Keywords:** mechanotransduction, matrix stiffness/deformation, shear stress, cytotoxicity

## Abstract

Integration of biophysical stimulation in test systems is established in diverse branches of biomedical sciences including toxicology. This is largely motivated by the need to create novel experimental setups capable of reproducing more closely in vivo physiological conditions. Indeed, we face the need to increase predictive power and experimental output, albeit reducing the use of animals in toxicity testing. In vivo, mechanical stimulation is essential for cellular homeostasis. In vitro, diverse strategies can be used to model this crucial component. The compliance of the extracellular matrix can be tuned by modifying the stiffness or through the deformation of substrates hosting the cells via static or dynamic strain. Moreover, cells can be cultivated under shear stress deriving from the movement of the extracellular fluids. In turn, introduction of physical cues in the cell culture environment modulates differentiation, functional properties, and metabolic competence, thus influencing cellular capability to cope with toxic insults. This review summarizes the state of the art of integration of biophysical stimuli in model systems for toxicity testing, discusses future challenges, and provides perspectives for the further advancement of in vitro cytotoxicity studies.

## 1. Introduction

The ongoing development of experimental setups for in vitro toxicity testing, in particular the advent of organ-on-a-chip models [1,2,3,4,5,6,7,8,9,10,11,12,13], opened new dimensions in deciphering cytotoxicity processes. With the introduction of biophysical stimulation, for instance in microfluidics systems or 3D structures, an additional degree of complexity is added to the biochemical reactions triggered by the compounds of interest. Several recent reviews highlight the importance and the need of organ-on-a-chip toxicological models, as well as the advantages of 3D cell cultures [4,5,6,7,8,11,12]. Continuous quality improvement of the in vitro tests is essential in order to sustain the reduction of the use of animal models in toxicity studies. In agreement, the number of publications including organ-on-a-chip/microfluidics models is constantly rising, as well as the offer of commercially available devices [1]. The constraints between disciplines are becoming more and more blurred, and the systematic comprehension of the impact of physical-mechanical stimuli on cellular responses is an important prerequisite. In order to increase the similarity with in vivo exposure, novel approaches need to combine biomechanical signals (i.e., shear stress, mimicking the behavior of extracellular fluids; variation of extracellular matrix (ECM) composition, stiffness, pressure or strain) with chemical and biochemical challenges (i.e., pharmaceuticals, toxins, nanomaterials, and endogenous signaling molecules like hormones or cytokines).

In the body, cells constantly integrate tensional forces due to the presence of neighboring cells and movement of body fluids [14,15,16]. Dynamic biomechanical stimulation tunes cell surface area/spread as well as cell adhesion and orientation/alignment [17,18,19]. These phenomena are accompanied by the following molecular events:

*i)*  modification of membrane curvature/tension [20,21];
*ii)*  re-distribution of transmembrane proteins [22,23];
*iii)*  modulation of ionic fluxes through mechanosensitive ion channels [24,25,26,27];
*iv)*  deformation of cyto-nucleoskeletal elements [28,29]; and
*v)*  reorganization of intracellular organelles [30,31,32].

**Figure 1:**
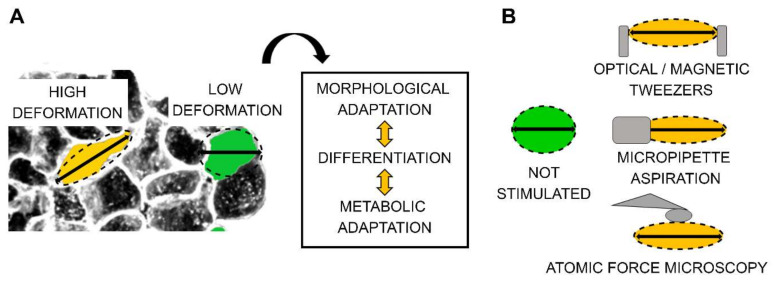
(**A**). Representative morphological adaptation resulting from the coexistence of cells in a multicellular organism. (**B**). Schematic representation of the procedures used to mimic cell deformation in vitro.

All these processes orchestrate the regulation between morphological adaptation, cell differentiation and metabolism (Figure 1A). In this line, toxicity can derive from loss of biomechanical compliance, both at macroscopic and microscopic levels. For example, it is known that intercalation of heavy metals like lead (Pb) and cadmium (Cd) into bones leads to a severe impairment of the bone mechanical endurance and increases the risk of fractures [33,34,35]. Similarly, at the single cell level, cytoskeleton-modifying agents like cytochalasin D or taxol can alter cell adhesion, morphology, and stiffness [36,37,38,39,40]. In vitro perturbation of cell mechanical environment can be reproduced with different approaches like optical/magnetic tweezers, micropipette aspiration, or atomic force microscopy (AFM) [21,41] (Figure 1B).

However, in-depth knowledge on the influence of biomechanical stimulation on cytotoxicity and accompanying crosstalk is far from being comprehensive. This is especially true when considering modulation of processes attributable to biophysical changes of the environment. To elucidate the intimate relationship between mechanosensory structures and toxicological targets, integrins serve as representative example. Cells need an anchoring apparatus to allow contact with the ECM and orientation in response to biomechanical stimulation [15,41]. The combination of α and β integrin heterodimers bridges the connection between the intracellular environment and different components of the ECM like laminin or collagen [42,43,44]. Intriguingly, it was recently highlighted how the physiological role of integrins could be extended toward the binding of viruses, bacteria, and parasites as well as being a target for several poisons [45]. In this line, food constituents and contaminants can interact directly with integrins. For example, the polyphenol resveratrol was described to bind to the hetero-dimeric alphaVbeta3 integrin in MCF-7 human breast cancer cells [46]. Similarly, the plasticizer bisphenol A can interact with the integrin β1 [47]. Bisphenol A also inhibits the attachment of human trophoblastic Jeg-3 spheroids to Ishikawa cells [48]. The molecular mechanism mediating these effects relies on the sensitivity of integrins to 17β-estradiol. Binding between the two is physiologically necessary to mediate the adhesion of blastocysts to the endometrium [49], albeit transforming the ECM-force transducers into potential targets for noxious compounds like endocrine disruptors.

The present work focuses on current knowledge about the integration of shear stress, ECM strain, and stiffness variation into cytotoxicity studies. Hence, we highlight the impact of biomechanical stimulation related to the extracellular environment on cell populations. The alteration of single cell biophysical properties attributable to toxicity (e.g., cytoskeletal alteration, motility, and migration/invasion) is beyond the scope of this work and was not included. For the selection of toxicity and cytotoxicity studies (chemical stimulation), we included data on cell viability/response derived from contaminants, drugs, and nanomaterials, as well as chemically induced modulation of inflammation and oxidative stress. Data published in the last 25 years were considered and key words included “shear stress and cytotoxicity/toxicity,” “stretching and cytotoxicity/toxicity” “biomechanical stimulation and cytotoxicity/toxicity,” “mechanical strain and toxicity,” “matrix/substrate/ECM stiffness and toxicity/cytotoxicity”. This approach allowed us to summarize observational and mechanistic studies and to identify the strength and the limitations of the integration of biophysics in cytotoxicity testing.

## 2. Substrate Stiffness 

In the body, as well as in an artificial cell culture environment, cells can be classified as suspension cells—growing and proliferating without the need to attach to a substrate—and as adherent cells. The latter have developed anchoring systems enabling the connection to the ECM [50]. ECM provides support and architectural organization necessary for tissue development in three dimensions, as well as a structurally organized microenvironment that contributes to extracellular compartmentalization and the generation of gradients of secreted molecules [51], nutrients, and even toxicants [8]. In addition to chemical cues, the ECM is defined by its biophysical properties and in particular by its stiffness (defined as resistance of an elastic body to deformation imposed by a force [52] and measured as elastic modulus or Young’s modulus). As summarized by Wang et al. [52], normal body tissues exhibit elastic moduli in the range of 100 Pa (brain tissue) to 100 kPa (soft cartilage) in contrast to 1 GPa of plastic vessels used for tissue culture.

### Modulation of ECM / Substrate Stiffness in Toxicological Context 

Cultivation of cells under the influence of differential substrate stiffness has been shown to tune cellular phenotype influencing spread and/or the formation of stress fibers [53,54]. Differentiation of muscular and neuronal cells is highly dependent on the stiffness of the surrounding environment [55]. Similarly, carbon nanotube scaffolds enhance functional performance in vitro of excitable cells like neurons and cardiomyocytes [56,57]. Differentiation of mesenchymal stem cells can be directed by ECM stiffness, with softer substrates triggering a more neuronal phenotype and harder substrates inducing progressively muscular or osteoblast-like differentiation [58]. Likewise, differentiation on patterned substrates, namely forcing cells into physical boundaries, can also drive toward a specific phenotype of mesenchymal stem cells [59,60]. Biophysical properties of the ECM are as important for physiological processes as for pathological ones. Loss of physical constraints can be associated with disease progression. For example, several studies underline an intimate relationship between cancer and ECM [61,62,63,64]. ECM stiffness was related to crucial parameters in cell resistance such as *i)* chemotherapeutics availability, *ii*) epithelial mesenchymal transition (EMT), and *iii*) regulation of oncogenic signaling pathways [65], thus highlighting ECM as an essential player in determining tumor progression [66,67]. Moreover, high-density tumor-specific ECM can hinder the T-cells proliferation and infiltration, without affecting cancer cells [68]. This provides crucial mechanistic insight in the role of matrix stiffness in sustaining cancer immune evasion. In line with the importance of physical cues in oncogenic progression, tumor cells can tune their sensitivity to chemotherapy in response to variation of the composition of the ECM. In 1999, Sethi et al. described reduced apoptosis in small cell lung cancer cell lines (H69, H510, H345) in response to classical chemotherapeutics (doxorubicin, cyclophosphamide, and etoposide) when cultivated on functionalized substrates like laminin, fibronectin, and collagen IV in comparison to standard culture conditions [69]. Looking for common targets mediating the response to physical and chemical stimuli, the transcription factors YAP/TAZ offer a clear example. Indeed, YAP was reported to affect 5-FU chemoresistance in colon cancer cells [70]. At the same time, YAP/TAZ are also regulated by biophysical cues, being sensitive to changes in cell shape as those deriving from adaptation to ECM stiffness or shear stress [71]. Hence, it is clear that mechanisms sustaining chemotherapy resistance and mechanotransduction can interplay with the same molecular pathways.

With respect to the integration of biophysical stimulation in cytotoxicity studies, variation of the ECM is the approach which was more extensively explored, as already excellently reviewed [8,72]. Some examples are provided in Table 1 to allow easier contextualization with the response to shear stress and substrate deformation (strain). In accordance to the well-established role of ECM in cancer research, many studies focus on the interaction with chemotherapeutic drugs (Table 1). However, response to environmental contaminants and uptake of nanoparticles (NPs) can also be modulated by substrate stiffness (Table 1). Recent transcriptome analysis performed on human vascular smooth muscle cells revealed that stiffness-sensitive genes are more conserved than stiffness-insensitive genes [73] making the research combining substrate topology and biophysical properties a promising approach also for future toxicological research.

## 3. Shear Stress 

Shear stress describes “the force per unit area created when a tangential force acts on a surface” [83]. Similarly, Hahn and Schwartz provided the definition as “the frictional force per unit area that a fluid exerts as it flows over a surface” [84]. The shear stress concept is used in biological context to define the biomechanical stimulation deriving from the movement of body fluids parallel to the tissue/cells of interest. It is expressed either according to the SI system in N/m² (equivalent to Pascal) or as dyn/cm^2^. Shear stress differs massively within the body, and so does the response of the individual cell types to this stimulus; typically, shear stress ranges from the high flow of the arterial and venous circulation (up to 30 dyn/cm^2^) to micro flow fluxes in the interstitial compartments (0.1 dyn/cm^2^ and below) [85,86]. Many studies integrate shear stress experiments in relation to the pathophysiology of the cardio-vascular system [84,87]. Recently, also in cancer biology the importance of mechanotransduction rising from movement of extracellular fluids is becoming more and more prominent and strongly contributes to the comprehension of the complex gain and loss of function characterizing the metastatic spread [63,66,85,88].

### 3.1. Modulation of Shear Stress in Toxicological Context

#### 3.1.1. Endothelial Cells 

Endothelial cells and in particular human umbilical vascular endothelial cells (HUVECs) are among the best characterized models for the combinatory effects with shear stress stimulation. This is attributable to the substantial impact of mechanotransduction on the homeostasis of vascular endothelial cells [84,87]. In line with the importance of shear stress for the pathophysiology of this cell type, a recent review from Bowden and colleagues described in detail specific methods for the integration of the biomechanical stimulation in the experimental layout [89]. Endothelial cells are dependent on shear stress for the handling of reactive oxygen species (ROS) and for the suppression of pro-inflammatory cytokines [90]. Furthermore, they can interpret shear stress decrease or transition from laminar to oscillatory flow as pro-inflammatory signals [91,92]. Similarly, adhesion of THP-1 monocytes on TNF-α-activated HUVECs can be regulated in a shear stress dependent manner [93]. In vascular endothelial cells, responsiveness to shear stress is ensured by a sophisticated apparatus. This relies on several components such as the platelet endothelial cell adhesion molecule (PECAM)-1, vascular endothelial cell cadherin (VE-cadherin) and vascular endothelial growth factor receptor 2 (VEGFR2) [94,95]. Mechanosensitive pathways allow endothelial cells to tip the balance between pro- or anti-inflammatory processes and to manage oxidative stress [83,96]. For example, expression of intracellular adhesion molecule 1 (ICAM-1) and the vascular cell adhesion molecule (VCAM-1) can be regulated by shear stress and by pro-inflammatory signals [97,98]. Similarly, atherosusceptible (low, 2 dyn/cm^2^) shear stress combined with TNF-α enhances the endoplasmic reticulum stress response that in turn regulates the expression of VCAM-1 and promotes monocyte recruitment [99]. The highly specialized mechanosensory apparatus also enables vascular endothelial cells to tune their metabolism according to the extracellular flow [100,101,102]. Flow-dependent metabolic adaptation is also mirrored in the capability to detoxify xenobiotics at endothelial level. In 2013, Wang et al. described that laminar flow (atheroprotective) can activate the Pregnane X receptor (PXR), whereas the oscillatory flow suppresses its activity [103]. These data are in agreement with the interpretation that endothelial cells can not only adapt to shear stress, but also differentiate among flow types [84]. Antagonistic responses might be triggered by shear stress in endothelial cells according to flow- or force [99,104] influencing the crosstalk of biophysical stimuli with cytotoxicity cascades (Table 2).

##### Interaction of Endothelial Cells and NPs

Among the combinatory toxicity studies using endothelial cells, several examples describe the effects of nanoparticles in presence or absence of shear stress. This is a very plausible experimental scenario since NPs are widely used as carriers for drug delivery [113,114] or in the food industry [115,116]. After entering the blood circulation, e.g. by intravenous injection, they are distributed in the circulation and via mediation of the vascular endothelium might enter adjacent tissues [117]. Fede et al. described in two consecutive papers an increased resistance of HUVEC endothelial cells when exposed to gold nanoparticles (Au-NP) in presence of shear stress as compared to static conditions [106,108]. In more detail, after exposure of the cells to 13 ± 3 nm NPs, they observed decreased cytotoxicity and nanoparticle uptake when combined with flow stimulation (5 µL/min) in comparison to static incubation [106] and confirmed the results also with bigger particles (24 ± 8 nm) [108]. Similarly, reduced uptake of Au-NP in the same cell type was obtained following shear stress pre-conditioning (24 h pre-incubation, 10 dyne) and incubation under flow conditions [107]. An explanation for the decreased NPs internalization under mechanical stress might be a reduced endocytic activity that, under shear conditions, serves to accomplish surface area homeostasis [17,118,119]. Moreover, Gomez-Garcia et al. described that co-localization of fluorescent NPs with HUVEC cells is modulated by the flow rate (0.1–0.8 Pa shear stress) and that cells respond differentially to laminar or disturbed flow preconditioning (0.1 Pa shear stress for 24 hours prior to exposure to 200 nm particles for 30 min) [109]. In 2011, Kim and co-workers published a systematic study of the toxic effects of mesoporous silica nanoparticles (MS NPs) on endothelial cells. To this aim, the authors investigated the influence of increasing shear stress stimulation (6.6–3.3–0.5 N/m^2^, 2h) on the toxicity of MS NPs (200 µg/mL, no effect level in static condition) and observed a flow dependent increase of the cytotoxic potential of the NPs measured via MTT assay. Moreover, they compared the effect of polyethylene glycol (PEG)/trimethyl silane-(TMS)-modified fluorescent MS NPs with non-coated fluorescent MS NPs and applied fluid shear stress of 3.3 N/m^2^ and 6.6 N/m^2^ for two hours. Functionalization significantly reduced the adhesion properties of the MS NPs, as well as the cytotoxicity in combination with both shear stress protocols, thus suggesting that the response of endothelial cells is equally influenced by the biomechanical stimulation as well as the chemical properties of the particles [105]. Similar conclusions were reached by Kusunose et al. describing the binding of NPs functionalized with NGR (targeting aminopeptidase N) and VHP (targeting VCAM-1) to HUVEC cells; also in this case, functionalization and physical status (static incubation or in shear stress) interplayed in determining the final outcome [120].

##### Interaction of Endothelial Cells with Pharmaceuticals

Combinatory studies on endothelial cells including shear stress can be of relevance for several compounds reaching the bloodstream. Feng et al. established a microfluidic system that enables users to test the cytotoxic potential of drugs in combination with increasing fluid shear stress. With this setup, they tested the effects of Vandetanib (inhibitor of the vascular endothelial growth factor receptor, 8 µM) in combination with flow rates ranging from 0.01 Pa to 0.09 Pa. In static conditions, HUVEC incubated with the drug displayed no difference in comparison to untreated controls. However, co-incubation with biomechanical stimulation increased cell morphological variation, apoptosis rate and ROS production in a “flow-dependent manner,” thus enhancing the response of the endothelial cells to Vandetanib [110]. Similarly, sustained shear stress (24 h, 5 dyn/cm^2^) enhanced the sensitivity of HUVEC monolayers to the anticancer drug doxorubicin (1 µM) in comparison to static incubation and to lower shear stress [111]. A similar reaction could be obtained by the co-incubation of doxorubicin with the small molecule Yoda-1 [111], which in turns activates the protein Piezo-1, the latter being crucial in sustaining cellular sensitivity to biomechanical stimulation [121]. The possibility to cultivate endothelial cells under more physiological conditions, namely using perfusion systems, is also crucial for the creation of more efficient blood–brain barrier (BBB) cell culture models [122]. This was recently confirmed by Park et al. taking advantage of human induced pluripotent stem (iPS) cell technology for the differentiation of brain-like microvascular endothelial cells (iPS-BMVECs) in a BBB chip system. They demonstrated how cultivation under flow could ameliorate the performance of the barrier in comparison to the static model. This approach has great importance on toxicological research. For instance, only through the integration of the microfluidics system it was possible to reproduce in vitro the behavior of the drug citalopram and to align to previous in vivo data describing the function of the efflux pumps [123].

#### 3.1.2. Non-Endothelial Cells 

Literature analysis points toward a cell-type specific integration capability for physical and chemical signals, tightly related to the functional ancestry of the original tissue. In addition to endothelial cells, shear stress stimulation can be of relevance for the physiological function of many organ systems like for instance the gastrointestinal tract [124,125,126] or the kidneys [3,9,10,127,128]. In general, combinatory studies report an amplification of the biological response compared to static conditions. This can be the result of an enhanced metabolic competence triggered by shear stress or a modification of the uptake profile. According to the test system, the outcome can be an increased or decreased toxicity.

**Hepatic cells**: Even though hepatocytes are separated from the sinusoidal blood by endothelial cells, they also possess highly functional membranes that mediate the exchange with fluids [129]. In line, cultivation of liver cells under shear stress is efficient in promoting their metabolic capability. Rashidi and co-workers [130] described how hepatocyte-like cells can be cultivated under flow (2.9 × 10^−5^ and 4.7 × 10^−5^ dyn/cm^2^) and can regulate the activity of CYP1A2 enzymes in a shear stress dependent manner [130]. These data confirmed previous observations that related shear stress stimulation with enhanced metabolic competence of hepatocytes in vitro. Xia and colleagues described in 2009 a bioreactor for the prolonged maintenance of hepatocytes in culture and observed that cells responded to the flow cultivation (0.03 mL/min or 5.6x10^-4^ dyn/cm^2^) with higher albumin secretion and urea production in the perfusion system, as well as with a time-dependent enhancement of the cytochrome P450 activity (measured with the 7-ethoxyresorufin-*O*-deethylation –EROD- assay). This phenotype was accompanied by an increased susceptibility to acetaminophen (25 mM APAP) for cells incubated in the bioreactor in comparison to static controls [131]. A similar outcome, with enhanced metabolism and respective sensitivity to different hepatotoxicants (APAP, ketoconazole, diclofenac, chlorpromazine, flutamide, and quinidine) was obtained in a high-throughput system [132]. In addition, Yu and colleagues described the potential of a liver-on-a-chip system integrating 3D rat hepatocytes spheroids in a perfusion culture platform for the discrimination of the difference between acute (APAP 0.1–10 mM) and chronic toxicity triggered by diclofenac (1–100 µM, MTS Assay) [133]. Similarly, cultivation of 3D MT of rat hepatocytes under gravity driven flow, induced a more efficient activation of cyclophosphamide to cytotoxic metabolites possibly due to enhanced medium exchange, nutrient supply and waste removal [134].

**Intestinal cells**: Intestinal lumen is characterized by continuous flow and shear stress stimulation. Accordingly, several bioreactors have been described to improve differentiation of intestinal cells in vitro [124,126]. Biomechanical stimulation was described to have an impact also on therapeutic protocols. Lou et al. described for instance increased susceptibility of colon cancer cells (T84 and SW480) to radiation in presence of prolonged shear stress stimulation (24 h, 12 dyne/cm^2^) and attributed this effect to a modulation of the integrin β1/FAK/Akt and cortactin pathways [135]. More difficult is the interpretation of the results, when considering the response of cancer cells in comparison to non-transformed ones. Colon adenocarcinoma cells (HT-29) display differential sensitivity to combined shear stress (1 dyn/cm^2^) and pro-oxidant challenge (mycotoxin altertoxin II, 1 µM) in comparison to non-transformed human colon epithelial cells HCEC. Intriguingly, HCEC cells responded efficiently to single treatments, but were unable to cope with combined physical-chemical stress. On the other side, the cancer cells presented a higher activation threshold for the single stimuli and maintained in return the capacity to integrate the combinatory challenge [136]. Kang et al. described for HT-29 cells a synergistic pattern between chemical and physical stimulation with an increased uptake of doxorubicin in presence of shear stress (0.5 dyn/cm^2^) and a boost in the cytotoxic potential [137]. Similarly, cultivation of intestinal HCT-116 in dynamic conditions incremented sensitivity to the chemotherapeutic 5-FU (0.1-0.5 mM) in comparison to static controls [138].

**Additional cell models**: Nephritic cells, like hepatic and intestinal cells, can be also cultivated in perfusion systems [3,9,10]. Cultivation under flow enhances the expression of junctional proteins like ZO-1 and occludin, thus increasing the epithelial barrier function. This approach allowed to identify differential toxicity of gentamycin given in a single bolus or a chronic regime [139]. Moreover, flow conditions (2–0.5 dyn/cm^2^) modulate the cytotoxicity of cyclosporine A (30 µM) in proximal tubule epithelial cells (PTECs) [128]. For human lung adenocarcinoma cell line, A549 and pancreatic adenocarcinoma cells PANC-1 shear stress induced no toxicity per se (0.5 dyn/cm^2^), but enhanced the cytotoxic potential of doxorubicin (1 µg/mL, MTT assay) [137]. Spencer and Backer described in MDA-MB-231 cancer cells a complex relationship between pharmacological treatment (paclitaxel 0.1–100 μM), ECM functionalization and shear stress (0.5 dyn/cm^2^) in modulating metastatic cell adhesion [140]. In line with the idea that cells can integrate shear stress and chemical stimulation, the response of C2C12 myoblasts to positively loaded nanocarriers was more affected by biomechanical stimulation than the responses to negatively and neutrally loaded vehicles [141]. In NIH/3T3 fibroblasts, shear stress enhanced toxicity of metals (CuSO_4_ and TINO_3_) compared with static incubation and cells displayed not only a concentration dependent cytotoxicity, but also a biomechanical-dependent responsiveness, with progressive increase of cytotoxicity and morphological alterations with the rise of the flow intensity [142].

In addition to the experimental observations provided by the studies, more and more intriguing questions rise around the mechanisms sustaining these events. Even though it is recognized that mechanotransduction regulates gene transcription [143], molecular effectors governing these cascades and down-streaming pathways are continuously discovered. Particularly transcription factors are described to be sensitive to shear stress. For example, in the resolution / promotion of inflammation, NF-ĸB [144] can be regulated also via biophysical stimulation. Disturbed flow can trigger a sustained NF-κB activation [145], whereas steady laminar flow induced anti-inflammatory pathways via NF-ĸB -COX2-prostaglandin E2 (PGE2) to suppress the effects of tumor necrosis factor-α (TNF-α) [146]. This implies a central role for shear stress in sustaining pro- and anti-inflammatory cascades. Additional examples for shear-stress sensitive transcription factors are discussed in Section 6.3 in relation to metabolic adaptation pathways.

## 4. Mechanical Substrate Deformation/Strain

Adaptation to ECM changes or tensile forces belong to well-known physiological cell responses. For the musculoskeletal apparatus, as well as for vascular endothelium, the central importance of mechanotransduction is known since decades, playing an essential role in muscle cells differentiation and physiology [147,148,149] or in the maintenance of bone structure integrity [150,151]. Along this line, cyclic strain is a powerful tool to drive/ameliorate cell differentiation [152,153]. For the application of uni-axial or multi-axial strain to cell populations, a reproducible force needs to be applied to the surface hosting the cells. A constant deformation of the ECM and of the neighboring cells is obtained in a static or cyclic fashion [154,155,156]. This type of stimulation can be regarded as combination of tensile and shear stress [118] and cells can be cultivated with deformation frequency up to 10 Hz [157,158].

### Modulation of Mechanical Strain in the Toxicological Context 

Combinatory toxicity studies applying strain are relatively rare in comparison to stiffness variation of the ECM or application of shear stress. These studies focus primarily on cellular nanoparticle accumulation, cytotoxicity or inflammatory cell responses. In 2014, Freese and colleagues compared the effect of silica NPs on HUVEC cells in presence or absence of biomechanical stimulation (cyclic equi-biaxial stretch, 1Hz, 5% elongation, MTS assay) [159]. This type of stimulation was chosen to model exposure of endothelial cells to pulsatile blood flow. Intriguingly, even if the cytotoxicity profile was quite similar between static conditions and biomechanical stimulation, the latter decreased the uptake of silica NPs without altering cell stress or increasing the secretion of pro-inflammatory mediators [159]. In contrast, augmented particle accumulation compared to non-stretched control cells, was observed by Hu et al., 2015 [160], after exposure of bovine aortic endothelial cells to polystyrene NPs, applying cyclic equi-biaxial strain at 5–15% elongation. Increased accumulation of quantum dots was accompanied by a parallel effect on the secretion of IL-8 by human keratinocytes (HEK) during strain (cyclic strain, 10% elongation) [161]. In addition, when applying unidirectional cyclic strain (10% elongation, 0.2 Hz) together with silica NPs to alveolar epithelial cells (NCI-H441), Huh et al. observed an enhanced production of reactive oxygen species in a lung-on-a-chip model. In addition, the number of cells that had accumulated NPs were reported to increase under mechanical stimulation [162].

Recently, experiments combining biomechanical stimulation (cyclic equi-biaxial strain, 15% area expansion, 0.25 Hz) with exposure to 25 nm amorphous colloidal silica NPs in A549 cells as model for type II alveolar epithelial cells revealed alterations in gene expression resembling an inflammatory cell response in comparison to the single stimuli. In this study neither cytotoxicity (LDH-assay) nor increased accumulation of NPs were observed [163]. These data indicate also in case of strain the capability of cells to integrate mechanical response and chemical stimuli toward a specific outcome. Cyclic strain can be also used to reproduce mechanical damage in vitro. Feng and colleagues demonstrated that propofol suppresses High Mobility Group Box-1 (HMGB1) production by mechanical strain in vivo as consequence of the ventilation process in the pulmonary tissue and reproduced similar results in vitro by applying cyclic stretch to mouse lung vascular endothelial cells [164]. Similarly to the lungs [165], also the skin is constantly challenged by biomechanical stimulation [166]. The mycotoxin deoxynivalenol (DON) (0.1–10 μM) was used to study the potential of a ribosomal inhibitor to influence biomechanical response in A431 epidermoid squamous cell carcinoma (0.5 Hz, 15% substrate deformation). Indeed, in control conditions, A431 cells showed compliant response to the deformation protocol with increase of tubulin network and lysosomal signal. These responses were impaired by pre-incubation with DON, even at sub-cytotoxic concentrations (measured with WST-1 assay), implying that cellular adaptation to biomechanical stimulation can per se be used to monitor cytotoxicity [32]. Moreover, cell “training” with the application of strain prior to addition of the mycotoxin resulted in an increased tolerance to the cytotoxic insult [32]. Indeed, biomechanical stimulation can be applied concomitantly to the chemical stimulation or sequentially thus offering multiple possibilities in the experimental plan and in the interpretation of the results. 

## 5. Integrating Biophysical Stimulation in Experimental Layout and Data Analysis

Taking as a starting point that cells are equally able to integrate physical stimulation as well as chemical and electrical signals, the classical concept of “dose” can also be translated to biophysical stimulation. In this light, biomechanical stimulation (i.e., shear stress, strain or compression) can be tuned and cellular responses often follow the classical “dose-response” or “force-response” paradigm. As such, excessive mechanical stimulation can also result in cell-tissue injury; for example, excessive respiratory muscular stress during artificial ventilation causing self-inflicted lung damage [167] and compression combined with hypoxia and alteration of skin mechanical properties leading to formation of pressure ulcers [168]. Decades of research were invested to shed light on mathematical models suitable to describe synergism and antagonism related to combinations of chemicals [169,170]. The integration of biophysical stimulation and the grouping of signals of physical and chemical origin represents an additional demanding task. Unfortunately, studies presenting toxicological endpoints combined with biomechanical stimulation often diverge massively in the experimental layout and this jeopardizes the possibility to draw common conclusions. Indeed, many systems are custom-made and the experimental challenge starts already with the design of the device for the biomechanical stimulation and the definition of appropriate cell culture conditions [165,171,172]. However, these systems maintain the advantage to tailor every detail of the experimental need. For the selection of technical parameters, many studies strive for biophysical settings (shear stress, stiffness, strain) resembling the in vivo parameters. Even if these reference values are important, complementary data about the performance of the specific systems are also necessary. Exactly like for the dose-response, mechanical stress-response curves help to define the capacity and the limits of a model in vitro. In-depth knowledge about physical cues would contribute to ease the comparison of results obtained with different instruments or cell lines. Moreover, it would allow to apply more refined statistical analysis in the comparison of the results and might additionally improve reproducibility of research results.

**Figure 2:**
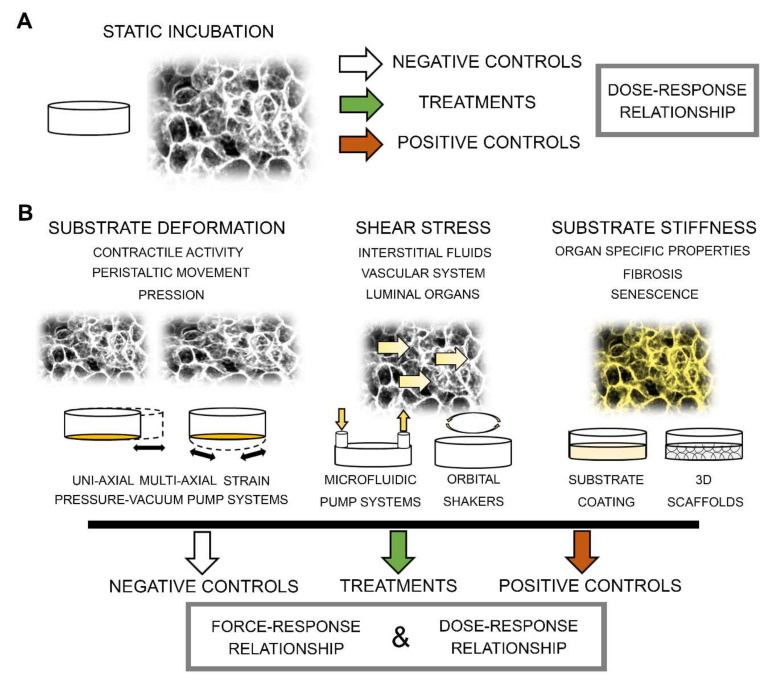
(**A**). Representative experimental layout in static conditions. (**B**). Representative experimental layout in presence of biomechanical stimulation to mimic specific pathophysiological processes.

Related to data interpretation, artifacts can easily originate in case the cells are not cultivated under comparable conditions. Making a parallel to “classical mixture toxicology,” for the evaluation of synergism, antagonism or additive effects, dose-response curves for the single compounds are also necessary [170]. For chemical-physical combinations this includes finding matching conditions for vessel size, surface material, and medium/volume ratio. In comparison to a classical experimental layout in static incubation, this results in a rapid increase of the experimental conditions, and eventually of the costs (Figure 2).

In an attempt to reduce these issues, several research groups optimized the application of biomechanical stimulation in systems integrating a multiwell-plate format. Following these strategies, there are examples for the modulation of substrate stiffness [77] or shear stress [140]. An additional possibility includes the use of rather common laboratory equipment like in the orbital-shaker method for the cultivation of plates under shear stress [173]. Moreover, commercial systems are more and more diffuse and offer support and standardization of the experimental conditions [174]. Additional technical details as well as pros and cons in the use of microtiter well-plates and microfluidics systems for drug discovery can be found in Regnault, Dheeman, and Hochstetter [175].

## 6. Integrating Biophysical Stimulation in Toxicokinetics Models 

The creation of progressively more accurate and complex systems also expands the possibility to bridge the gap between in vitro and in vivo studies for the generation of toxicokinetics data. Several working groups devoted their attention to the integration of organ-on-a-chip microfluidic models in the pharmacokinetics analysis. Numerous recent reviews summarize detailed information about the intestinal tract [176,177], liver [5,178] or multi-organ-systems [177,179,180,181,182,183]. Indeed, cell cultivation in presence of biomechanical cues can help to reproduce in vitro processes that typically characterize the ADME in vivo.

### 6.1. Absorption

Among the most common in vitro approaches to model intestinal absorption is the cultivation of Caco-2 cells on permeable supports, typically following a 21-days differentiation protocol [184]. The model can be expanded to include additional cell types like mucus-secreting goblet cells and M cells [185] or via the integration of biomechanical stimulation. Experiments performed in presence of shear stress [186] or shear stress plus mechanical strain (substrate deformation) [126] can notably speed up and diversify the differentiation process promoting epithelial polarization and villi formation. This is accompanied by uptake/permeability performance comparable or superior to static differentiation or the Ussing chamber [126,186]. Modelling the absorption of NPs is even more challenging, even though of outermost relevance for the uptake of drug nano-carriers and nano-formulations at endothelial level. Under static conditions, NPs reach the cellular surface mainly driven by diffusion and sedimentation [187]. In comparison, high shear stress reduces the amount of particles delivered to the cell surface as deposition requires escape from the liquid flow [188,189], a phenomenon that does not apply to small molecules. Indeed, Charwat et al. described a biphasic behavior of shear dependent NPs uptake by HUVEC cells. NPs uptake was dose-dependent parallel to the increase of the flow stimulation up to a critical value (2.25 μL min^−1^ or 4 dyn cm^−2^). At a higher mechanical load, NPs absorption decreased with inverse relation between concentration and force [190].

### 6.2. Distribution

Cultivation of cells while integrating biomechanical stimulation allows to refine the description of distribution processes. It is worth noticing that some organ-on-a-chip devices on purpose exclude appreciable shear stress with the idea to reduce potential interference of physical stimulation with cell physiology, albeit maintaining chemical/medium exchange [191,192,193]. Microfluidics and 3D structures can be combined to explore the formation of physical and chemical gradients [110,194,195], as well as to study the modulation of barrier systems like the Blood Brain Barrier [90,123,196,197]. For example, primary porcine brain microvascular endothelial cells (PBMEC) align in response to shear stress and increase expression of junctional proteins (ZO-1) as well as the efflux ratio of the antineoplastic agent mitoxantrone [198]. This infers for increased functional performance of the BBB model in comparison to static condition. Sung and colleagues described already in 2010 a microfluidic device for PD/PK where three chambers allowed to reproduce the relative blood flow distribution to selected organs [138]. Drug-distribution profiles in the heart, lung and adipose tissue were obtained also thanks to microfluidic sandwich-devices designed to allow sequential passage through intestinal, endothelial, hepatic and breast cancer cells [199].

### 6.3. Metabolism

Several studies provide mechanistic insight in the potential of biomechanical stimulation to regulate metabolism. This includes the adaptation of basal metabolic competence in health and disease [200,201,202], mitochondrial function [100,202], autophagy [203,204], as well as xenobiotics metabolism. Shear stress regulates cytochrome (CYP) P450 activity in hepatic cells (Section 3.1.2 [130,131]). Likewise, endothelial cells can tune arachidonic acid metabolism via CYP P450 epoxygenases in response to shear stress and cyclic substrate deformation [205,206]. HUVEC cells increase expression of CYP1A1 in response to laminar shear stress [207,208] through a mechanosensitive translocation of the aryl hydrocarbon receptor (AhR [209]). Moreover, connection between mechanotransduction and metabolism can be achieved by means of the transcription factor Nrf2 (nuclear factor-E2 p45-related factor (Nrf) 2). Nrf2 connects mechanosensing to cell antioxidant capacity, and while mediating response to oxidative insults, it is also sensitive to mechanical triggers [92,210]. Nrf2 regulates phase II detoxifying and oxidative stress enzyme genes and plays a paramount role in xenobiotics metabolism [211,212]. In absence of a pro-oxidative stimulus or mechanical stress, Nrf2 is normally bound to its scaffolding protein KEAP-1, thus blocking the transcription of the Antioxidant Responsive Elements (ARE) in the DNA [211]. In presence of electrophiles and reactive oxygen species, oxidation of the cysteine residues connecting KEAP-1 to Nrf2 determines a release of the transcription factor and respective nuclear translocation [210]. Similarly, mechanical regulation of Nrf2 was also observed as response to shear stress: this effect was initially related to endothelial sensitivity to biomechanical stimulation [92,213,214]. However, similar mechanisms were recently described also for intestinal cell models like HT-29 and human colon epithelial cells (HCEC) [136], thus opening totally new perspectives in the interpretation of toxicologically relevant events like food-borne exposure to xenobiotics and the interplay with intestinal cells function and motility. Similarly to Nrf2, the transcription factors Klf (Krüppel-like factors) have been described as regulators of cell metabolism in response to physical stimulation. Klf2 modulates the effect of Nrf2 and its target genes [215] in endothelial cells and mediates metabolic adaptation in response to mechanical stimulation [216].

### 6.4. Excretion

In the excretory function, kidney epithelial cells are constantly challenged by shear stress and substrate deformation [217]. Hence, biomechanical cues exert profound impact on their physiological regulation and cells can tune the activity of efflux pumps and transport mechanisms in response to shear stress [128,218]. In addition, renal proximal tubule epithelial cells increase the expression of tight-junctions in response to substrate topology and shear stress [219]. Similarly, hi-PS-derived podocytes integrated in an organ-on-a-chip platform present enhanced functional features in comparison to static cultures and allowed to reproduce in vitro filtration processes as well as drug-induced proteinuria [220].

## 7. Conclusions and Future Perspectives

Although it is well documented that biophysical stimulation can trigger directly cell morphological adaptation, how this finally reflects on cellular pathophysiological status remains a fundamental question. The creation of novel in vitro test systems thanks to biophysical approaches offers novel exciting opportunities. Together with multi-omics analysis and high-end imaging technologies, it promises to significantly boost our understanding of disease and toxicity mechanisms. This approach is central to promote the creation of model systems with higher predictive value and will contribute to progressively reduce animal testing. Regardless of the type of biomechanical stimulation, being either matrix stiffness variation, shear stress, or matrix stretching, such stimuli deeply impact cell responses to toxicants. The elucidation of the signaling pathways involved in the combinatory response to physical and chemical stimulation will be the basis for further understanding this crosstalk. Integrative responses occur directly, for instance when mechanical cues and toxic compounds share the same molecular targets. However, synergism or antagonism might develop indirectly through complementary modulation of signal transduction pathways (e.g., through mechano-regulated transcription factors). Prerequisite for the transition from static toward dynamic models is the comprehension of mechanisms transforming movement into biochemical pathways. This has the potential to greatly enrich the quality of our data and is crucial also for the creation of new toxicokinetics models. In parallel to the groundbreaking discoveries on the importance of mechanical stimulation in cell pathophysiology, integration of biophysical approaches in cytotoxicity profiling promises to greatly enlarge the possibilities in the performance of toxicity studies in vitro. Due to the complexity and the technical needs of the experimental setups, the data available so far are largely descriptive. However, this stage represents the necessary starting point for future research. Molecular mechanisms sustaining the abovementioned observations are just starting to be elucidated and represent an upcoming challenge for the scientific community.

## Figures and Tables

**Table 1:** Overview of publications describing a modulatory role of stiffness on cell responses to chemical stimulation and diverse substrate stiffness. (RGD refers to the functionalization with arginine-glycine-aspartic acid; polyacrylamide is abbreviated as PA or PAA, polyethylene glycol as PEG)

Physical Stimulation Stiffness	Chemical Stimulation	Cell Model	Response	Reference
Collagen Type I or alginate	Doxorubicin, 5-Fluorouracil, Tamoxifen	Hepatocellular carcinoma Hep3B and Breast adenocarcinoma MCF-7	No major difference between normal 96-well plates and 3D. Tendency toward increased resistance in the 3D structures.	[74]
PEG + *RGD* 241 ± 19 Pa 637 ± 93 Pa 1201 ± 121 Pa	Paclitaxel	human epithelial ovarian cancer cell line OV-MZ-6 and ovarian serous adenocarcinoma cell line SKOV-3	Shape and size of spheroids dependent on the matrix (> Stiffness > compactness and < size). RDG-enhanced proliferation. 3D culture decreased sensitivity to drug.	[75]
Alginate hydrogels	Acetaminophen, Diclofenac Rifampin, Quinidin	Hepatocellular carcinoma HepG2 and Breast adenocarcinoma MCF-7	*Acetaminophen, Diclofenac*, 3D cultures are more sensitive than 2D, *Rifampin, Quinidine* similar toxicity.	[76]
Collagen-coated glass Polyacrylamide 1 kPa	Multiple chemicals Including: Cantharidin and Okadaic acid Taxol, Cytochalasin D, PD173074 Blebbistatin	12 cell types: 16HBE14o- A549; c2c12; hASC HEK293; hMSC L929; MDCKII; MLE12 NHBE; NHDF; NHLF NIH3T3; RLE6TN	*Cantharidin and Okadaic acid* attenuated cell growth on *soft substrates* *Taxol, Cytochalasin D, PD173074*, major effect on *rigid substrates.* *Blebbistatin* growth stimuli on *soft substrates* and inhibitory on *rigid substrates.* No effect at 20kPa.	[77]
Polyacrylamide 1.6-5.7 kPa	NP	bovine aortic endothelial cells (BAECs)	Internalization per cell increases at higher stiffness (100 nm carboxylated polystyrene nanoparticles).	[78]
Polyacrylamide 1.5 and 40 kPa	LPS and TNF-α	Human pulmonary artery endothelial cells (HPAEC) and human lung microvascular endothelial cells (HLMVEC)	↑ stiffness ↑ response (ICAM1/VCAM1 and fibronectin).	[79]
Alginate hydrogels & tissue-culture polystyrene Storage modulus (Pa) 343 ± 28/3041 ± 191	Acetaminophen, Acrylamide, Cadmium chloride, and quinidine	Human U-87 and U-251 glioblastoma, IMR-32 neuroblastoma, and HEK 293 cells	Cells in *soft* alginate matrices ↑ sensitivity in comparison to cells encapsulated in stiffer matrices or 2D. RhoA activity modulation restores the resistance.	[80]
Alginate hydrogels ±RGD Storage modulus 400 and 3500 kPa	Acrylamide and Cadmium	Glioblastoma cells U-87 and U-251	*Soft substrate* without RGD ↑ sensitivity	[81]
Polyacrylamide 1-4-25 kPa	Gemcitabine and Paclitaxel	Pancreatic cancer cells BxPC-3 and Suit2-007 AsPC-1 cells	Response to nucleoside analogue *gemcitabine* was unaffected. Stiffness < 4 kPa ↑resistance to *paclitaxel*.	[82]
Polyacrylamide 1–25 kPa	NP	Breast cancer cell lines MCF-7 & MDA-MB-231	Internalization efficiency increases at higher stiffness’s (pluronic PEG-based micellar nanoparticles).	[52]

**Table 2:** Overview of publications describing toxicologically relevant processes in presence of shear stress in endothelial cells. For comparison conversion is provided between cgs unit dyn/cm^2^ and SI units N/m^2^, equivalent to Pascal (Pa). 1 dyn/cm^2^ = 0.1 N/m^2^ = 0.1 Pa.

Physical Stimulation Shear Stress	N/m^2^ (Pascal)	Chemical Stimulation	Response	Reference
6.6–3.3–0.5 N/m^2^	6.6-3.3-0.5	Mesoporous Silica NP	Shear stress modulate cytotoxic potential.	[105]
5 µL/min (0.1 dyn/cm^2^)	0.01	Gold Nanoparticles (13±3 nm Ø)	↑ viability in microfluidic device Live/Dead Assay.	[106]
10 dyn 3 h 10 dyn 24 h pre-inc.	1	Gold Nanoparticles (80 nm Ø; 5 µg/mL; 9.67 × 10^8^ particles/mL) TNF-α 10 ng/mL	↓ AuNPs uptake with shear stress and ↑ anti-ICAM-1 AuNPs uptake with shear stress and TNF-α.	[107]
5 µL/min (0.1 dyn /cm^2^)	0.01	Gold Nanoparticles (13 ± 3 and 24 ± 8 nm Ø)	↑ viability in microfluidic device Live/Dead Assay.	[108]
0.1-0.2-0.8 Pa 0.1 Pa 24 h pre-inc.	0.1-0.2-0.8	Red Fluorophore-loaded carboxylate-capped NP (200 nm Ø)	Uptake dependent on the laminar or disturbed flow.	[109]
0.01-0.09 Pa	0.01-0.09	Vandetanib 8 µM (no toxicity static)	Shear stress + Vandetanib induced morphological changes, ROS and apoptosis rate (%). No effect for drugs and shear stress alone.	[110]
5 dyn /cm^2^	0.5	TNF-α 100 U/mL Doxorubicin 1 µM	Shear stress ↓ICAM-1 and VCAM-1 induced by TNF-α. Shear Stress ↑ toxicity of Doxorubicin.	[111]
2–12 dyn /cm^2^	0.2-1.2	TNF-α 0.3 ng/mL	2-4 dyn /cm^2^ ↑ VCAM-1; 12 dyn /cm^2^ ↓VCAM-1 expression induced by TNF-α. Triglyceride-rich lipoproteins and shear stress modulate TNF-induced VCAM-1.	[104]
2 dyn /cm^2^	0.2	Ivabradine 0.04 μM	Ivabradine treatment ↓VCAM-1, IL-6 and ROS induced by shear stress.	[112]
